# Effect of erythropoietin administration on proteins participating in iron homeostasis in *Tmprss6*-mutated *mask* mice

**DOI:** 10.1371/journal.pone.0186844

**Published:** 2017-10-26

**Authors:** Jana Frýdlová, Zuzana Rychtarčíková, Iuliia Gurieva, Martin Vokurka, Jaroslav Truksa, Jan Krijt

**Affiliations:** 1 Institute of Pathological Physiology, First Faculty of Medicine, Charles University, Prague, Czech Republic; 2 Laboratory of Tumour Resistance, Institute of Biotechnology, BIOCEV Research Center, Czech Academy of Sciences, Vestec, Czech Republic; 3 Faculty of Pharmacy in Hradec Králové, Charles University, Hradec Králové, Czech Republic; Lady Davis Institute for Medical Research, CANADA

## Abstract

*Tmprss6*-mutated *mask* mice display iron deficiency anemia and high expression of hepcidin. The aim of the study was to determine the effect of erythropoietin administration on proteins participating in the control of iron homeostasis in the liver and spleen in C57BL/6 and *mask* mice. Administration of erythropoietin for four days at 50 IU/mouse/day increased hemoglobin and hematocrit in C57BL/6 mice, no such increase was seen in *mask* mice. Erythropoietin administration decreased hepcidin expression in C57BL/6 mice, but not in *mask* mice. Erythropoietin treatment significantly increased the spleen size in both C57BL/6 and *mask* mice. Furthermore, erythropoietin administration increased splenic *Fam132b*, *Fam132a* and *Tfr2* mRNA content. At the protein level, erythropoietin increased the amount of splenic erythroferrone and transferrin receptor 2 both in C57BL/6 and *mask* mice. Splenic ferroportin content was decreased in erythropoietin-treated *mask* mice in comparison with erythropoietin-treated C57BL/6 mice. In *mask* mice, the amount of liver hemojuvelin was decreased in comparison with C57BL/6 mice. The pattern of hemojuvelin cleavage was different between C57BL/6 and *mask* mice: In both groups, a main hemojuvelin band was detected at approximately 52 kDa; in C57BL/6 mice, a minor cleaved band was seen at 47 kDa. In *mask* mice, the 47 kDa band was absent, but additional minor bands were detected at approximately 45 kDa and 48 kDa. The results provide support for the interaction between TMPRSS6 and hemojuvelin *in vivo*; they also suggest that hemojuvelin could be cleaved by another as yet unknown protease in the absence of functional TMPRSS6. The lack of effect of erythropoietin on hepcidin expression in *mask* mice can not be explained by changes in erythroferrone synthesis, as splenic erythroferrone content increased after erythropoietin administration in both C57BL/6 and *mask* mice.

## Introduction

Iron is present in many proteins and is indispensable for life. Quantitatively, about 75% of iron present in the human body is contained in hemoglobin, and lack of iron therefore predictably leads to anemia. Iron-deficiency anemia is the most common form of anemia worldwide [1], and in the vast majority of cases it can be treated by iron supplementation. However, a minor fraction of patients with iron-deficiency anemia do not respond to oral iron administration, and this iron-refractory iron deficiency anemia can be caused by mutations in the *TMPRSS6* gene [2]. *TMPRSS6* encodes a transmembrane serine protease, matriptase-2 (TMPRSS6), which is expressed almost exclusively in the hepatocytes. Available clinical and experimental data demonstrate that functional TMPRSS6 is necessary for the appropriate regulation of hepcidin expression [2–6].

Iron metabolism is controlled at the level of iron absorption from the diet and at the level of heme iron recycling by macrophages. Both processes are regulated by hepcidin—a small peptide synthesized in the hepatocytes [7–9]. Hepcidin, encoded by the *HAMP* gene, controls iron metabolism by blocking the passage of iron from the diet to the bloodstream at the level of iron export from the enterocyte; decreased production of hepcidin results in increased uptake of iron from the diet and iron overload [10]. The mode of action of hepcidin is the degradation [11] of ferroportin (FPN)–a transmembrane iron export protein which exports iron from the enterocytes and macrophages to plasma. Hepcidin thus controls not only the uptake of iron from the diet, but also–at least to a certain extent [12]–the reutilization of iron from senescent erythrocytes following their phagocytosis by macrophages in the spleen.

Because erythropoiesis is dependent on a sufficient supply of iron, it is not surprising that treatment of mice with erythropoietin (EPO) decreases hepatic *Hamp* expression [13,14]. Recently, the mechanism of hepcidin downregulation by increased erythropoietic activity has been partially solved by the description of erythroferrone (ERFE), a *Fam132b-*encoded protein produced by erythroblasts, which, after secretion and an as yet undefined interaction with putative hepatocyte receptor(s), decreases hepatocyte *Hamp* gene transcription [15,16]. Interestingly, in iron-refractory iron deficiency anemia patients, as well as in *Tmprss6*-mutated mice, this downregulation of hepcidin by ERFE is not functional, and hepcidin transcription does not respond to administration of EPO [17–20]. At present, it is not precisely known how the *Tmprss6* mutation can affect the response of *Hamp* expression to EPO. Based on *in vitro* experiments, it has been proposed that TMPRSS6 cleaves hepatocyte hemojuvelin [21], a protein critically important for hepcidin gene regulation [22,23]. Strong support for this proposal comes from the observation that the hemojuvelin (HJV)-dependent signaling pathway is upregulated in *Tmprss6*-mutated mice [24, 25]; on the other hand, no direct *in vivo* evidence for TMPRSS6-mediated HJV cleavage has yet been obtained [26]. Alternatively, since the downregulation of *Hamp* expression by EPO requires both functional TMPRSS6 and functional ERFE, it is theoretically possible that both proteins operate in the same pathway and that the proteolytic activity of TMPRSS6 modulates ERFE function [27]; however, recent evidence argues against this hypothesis, since recombinant ERFE is able to downregulate *Hamp* in hepatocytes isolated from *Tmprss6-/-* mice [28]. At the protein level, it has already been shown that plasma ERFE levels are increased in *Tmprss6*-/- mice in comparison with wild-type mice [28]; no data regarding the effect of EPO on ERFE protein synthesis in *Tmprss6*-mutated mice have so far been published.

The purpose of this study was to examine the effect of EPO administration to *Tmprss6*-mutated *mask* mice on the expression of key proteins participating in iron metabolism, and to verify some of the pathophysiological mechanisms which are proposed to compromise the appropriate response to EPO in these mice. We demonstrate that the synthesis of both ERFE and transferrin receptor 2 is intact in spleens of *Tmprss6*-mutated *mask* mice treated with EPO, and that ferroportin protein content is decreased in the spleens of EPO-treated *mask* mice in comparison with EPO-treated C57BL/6 mice. In addition, we demonstrate that lack of TMPRSS6 alters the cleavage of hemojuvelin at the hepatocyte plasma membrane.

## Materials and Methods

### Animals and Treatment

Breeding pairs of *mask* mice were obtained from the Mutant Mouse Resource Research Centers, USA. Breeding pairs were kept on an iron-enriched diet (Harlan Teklad, 2% carbonyl iron, TD.09521, iron content approximately 20 000 ppm), weaned *mask* mice were kept on a standard laboratory diet (iron content approximately 200 ppm). Littermates with intact *Tmprss6* genes (C57BL/6 mice) were used for comparison. All animal experiments were approved by the Czech Ministry of Education, protocol MSMT-1461/2015-5, and by Czech Academy of Sciences experimental project no. 25/2015. Animals were sacrificed by decapitation under halothane anesthesia at 12–18 weeks of age.

Male and female *mask* and C57BL/6 mice received an intraperitoneal injection of EPO (NeoRecormon 2000, Roche, 50 IU/mouse/day, diluted in 250 μl of saline) on four consecutive days and were sacrificed 24 hours after the last injection; control animals received phosphate-buffered saline (PBS).

### Sample Preparation and Immunoblotting

Spleen homogenates for ferroportin and ERFE determination were prepared in 150 mM sodium chloride containing 50 mM Tris, pH 8.0, 2 mM of EDTA, 1.0% of Igepal Ca-630 (Sigma Aldrich) and a protease inhibitor cocktail (Roche). Approximately 50 mg of spleen tissue was homogenized in 2 ml of buffer; after 1 hour agitation at 4°C, samples were centrifuged at 12 000 g and the supernatant was used for analysis. Spleen microsomes for ERFE and transferrin receptor 2 (TFR2) determination were prepared by homogenization of approximately 60 mg of spleen tissue in 2 ml of 250 mM sucrose in 10 mM Hepes, pH 7.4, containing 2 mM of EDTA and protease inhibitors (Roche). The homogenate was centrifuged at 8000 g for 20 minutes and the supernatant was centrifuged at 100 000 g for one hour. The resulting microsomal pellet was resuspended in 90 μl of 2% SDS in 25 mM of ammonium bicarbonate, final protein concentration was approximately 5 μg/μl.

For the determination of liver HJV, a plasma membrane-enriched fraction of liver homogenate was used [29]. A piece of liver (approx 200 mg) was homogenized in 2.0 ml of homogenization buffer (10 mM Hepes, pH 7.4, 250 mM sucrose, 2 mM EDTA and protease inhibitors). After 30 min on ice, the homogenate was centrifuged at 400 g for 10 min and the supernatant centrifuged at 3000 g for 15 min. The 3000 g pellet was then homogenized in 1 ml of 10 mM Hepes containing 2 M NaCl, centrifuged for 15 min at 3000 g and homogenized in 1 ml of 0.1 M sodium carbonate. After 1 h agitation at 4°C, the homogenate was centrifuged at 16 000 g, and the pellet homogenized in 1 ml of 10 mM Hepes containing 4 M urea. After 30 min on ice, the homogenate was centrifuged at 16 000 g and the final pellet washed in 10 mM Hepes, recentrifuged and resuspended in 100 μl of 2% SDS in 25 mM of ammonium bicarbonate. The protein content of this plasma membrane-enriched fraction was approximately 3–5 μg/μl.

Immunoblotting was performed under reducing conditions on 8% polyacrylamide gels. For ERFE, HJV and TFR2 determinations, samples were heated at 90°C for 10 min, for FPN determination, samples were heated at 50°C for 5 min. Proteins were determined by commercial antibodies. For ERFE determination, goat anti-myonectin, Santa Cruz SC-246567, was used at 1:200 dilution. As recently reported [30], this antibody detects ERFE in microsomes from EPO-treated animals as well as murine recombinant protein *in vitro*; subsequent analysis of spleens from EPO-treated *Fam132b*-/- mice confirmed that the antibody detects ERFE protein (S1 Fig). For the determination of HJV, R&D Systems AF 3634 antibody was diluted 1:1000; for the determination FPN and TFR2, Alpha Diagnostic International MTP1-A and TFR21-A antibodies were diluted 1:2000. Anti- Na+/K+ATPase α (ATP1A), Santa Cruz SC-28800, 1:10 000, and anti-GAPDH, Sigma G9545, 1:30 000, were used as loading controls. Secondary antibodies (anti-rabbit, 711-036-152, 1:40 000 and anti-goat, 705-036-147, 1:40 000) were from Jackson Immunoresearch. Blots were blocked with 5% milk for 1 hour, incubated overnight with primary antibodies in 5% milk and subsequently incubated for 2 hours with secondary antibodies in 5% milk. Proteins were visualized after reaction with LumiGLO (Cell Signaling Technology) on X-ray paper (AGFA CP-BU, Germany). Signal intensities were obtained using Image Studio Lite from LI-COR Biosciences, and normalized to loading control protein intensities.

### Real time PCR and iron determinations

Samples for RNA isolation were harvested into RNALater (Sigma Aldrich), RNA was isolated using Qiagen RNAeasy Plus kit and transcribed by Fermentas RevertAid kit (Thermo Scientific). Real-time PCR was performed on a Bio-Rad IQ5 instrument using Bio-Rad SYBR green reagents, primer sequences are given in S1 Table. Amplification was linear over the range of the experiment. Results are expressed as Δ CT values relative to *Actb* expression (Δ CT = *Actb* cycle threshold–target gene cycle threshold; the higher the graphed Δ CT value, the higher the expression). Iron was determined according to Torrance and Bothwell [31]. PNGase F treatment was performed according to the manufacturers protocol (New England Biolabs). Hemoglobin and mean cell volume (MCV) were determined on Mindray BC-5300Vet analyzer, hematocrit was determined by centrifugation in capillaries.

### Statistical analysis

Hematology parameters, tissue data and Δ CT values were compared by one-way analysis of variance, followed by Tukey’s multiple comparison test, using GraphPad Prism software. For the comparison of immunoblot signal intensities from different blots, signals normalized to loading controls were expressed as percentages of signals from control C57BL/6 mice (for ferroportin determination) or from EPO-treated C57BL/6 mice (for ERFE and TFR2 protein determination) on the same blot. The obtained percentage values from different blots were then compared by one-way analysis of variance, followed by Tukey’s multiple comparison test. Differences with p < 0.05 were regarded as statistically significant.

## Results

### EPO administration does not significantly decrease *Hamp* expression in *mask* mice

**Fig 1:**
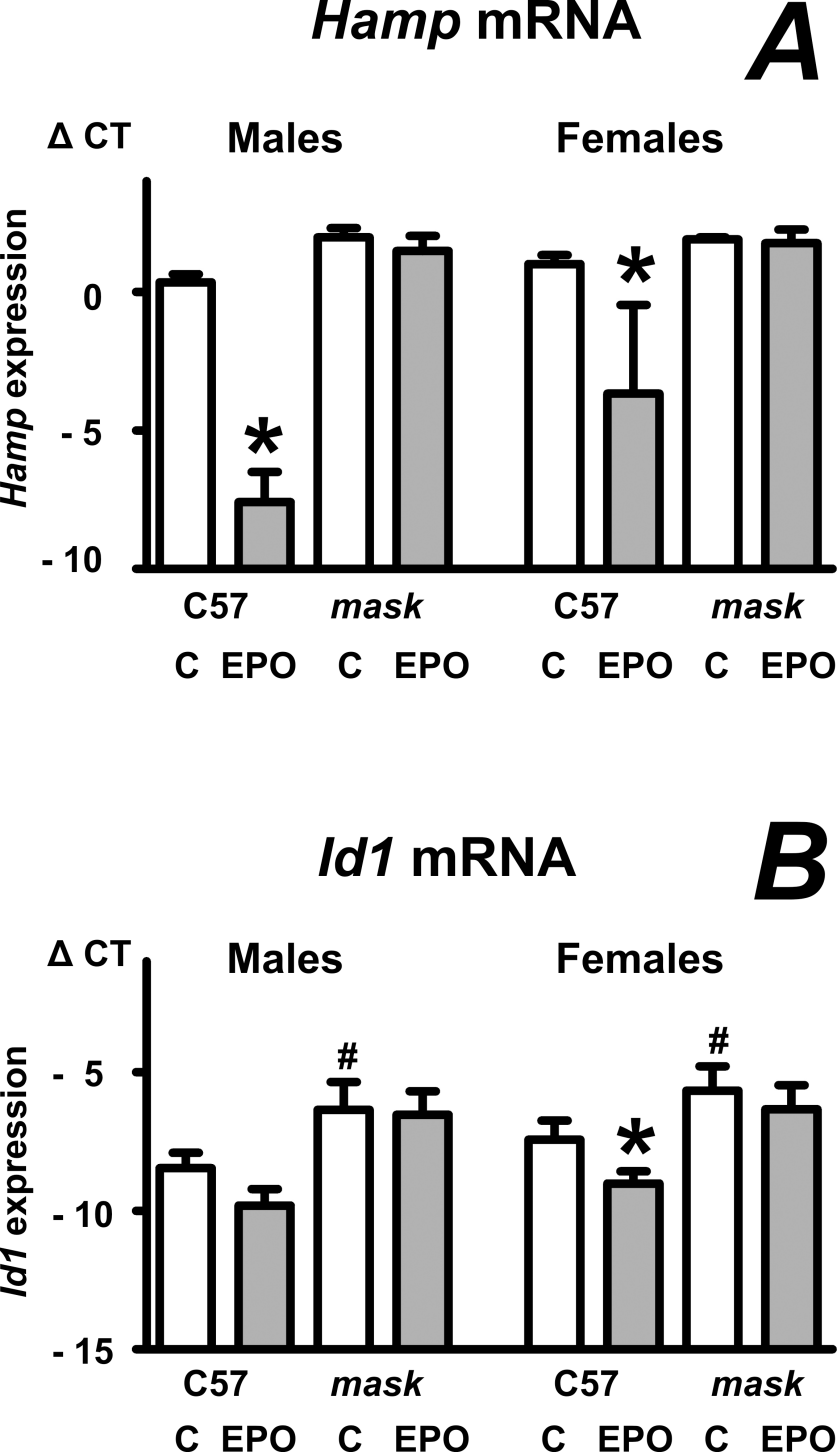
EPO administration does not decrease *Hamp* expression in *mask* mice. A and B: Effect of EPO administration (50 IU/mouse daily for four days) on liver *Hamp* and *Id1* expression in C57BL/6 (C57) and *mask* mice. Data are expressed as mean ± SD of Δ CT values obtained by real-time PCR (Δ CT = CT *Actb*–CT target), n = 4. Asterisks denote statistically significant difference between PBS-treated (C) groups and EPO-treated groups; hash signs denote statistically significant difference between C57BL/6 mice and *mask* mice (p<0.05).

Data presented in Fig 1A show that, in agreement with results from mice obtained by targeted disruption of the *Tmprss6* gene [18,20], EPO does not significantly decrease liver *Hamp* mRNA in *mask* mice, which were produced by N-ethyl-N-nitrosourea-induced mutation and lack the proteolytic domain of TMPRSS6 protein [3]. In agreement with *Tmprss6*-/- mice [20], *mask* mice do not significantly decrease liver *Id1* expression following EPO administration (Fig 1B). As also observed in *Tmprss6-/-* mice [18], EPO administration to *mask* mice for four days increased spleen size, but, in contrast to C57BL/6 mice, did not result in significantly increased hemoglobin levels (Table 1).

**Table 1:** Hematologic parameters in control and EPO-treated C57BL/6 and *mask* mice.

Group	Hb g/l	MCV fl	HCT %	Spleen wt mg	Liver Fe μg/g ww	Spleen Fe μg/g ww
**C57 male**	137±8	45±1	47±2	77±9	63±26	638±220
**C57 male EPO**	156±5*	47±1	54±1*	247±52*	43±18	224±52
***mask* male**	101±10#	28±3#	38±3#	93±21	37±17	582±211
***mask* male EPO**	103±16	27±3	40±5	188±48*	35±13	262±53
**C57 female**	141±6	45±2	47±2	80±12	99±20	816±442
**C57 female EPO**	168±21*	49±1*	58±9*	224±81*	54±33	286±138*
***mask* female**	115±9#	33±2#	41±3	84±12	40±6#	665±161
***mask* female EPO**	120±4	32±3	44±3	164±18*	58±21	317±52

EPO was administered to C57BL/6 (C57) or *mask* mice at 50 IU/mouse daily for four days. Values are expressed as mean ± SD, n≥7 for hematology data and n≥4 for tissue data. Liver and spleen nonheme iron is expressed as μg Fe/g wet weight. Asterisks denote statistically significant difference between EPO-treated groups and PBS-treated groups, hash signs denote statistically significant difference between C57BL/6 mice and *mask* mice (p<0.05).

### Expression of *Fam132b* and *Fam132a* genes is increased following EPO administration in C57BL/6 and *mask* mice

**Fig 2:**
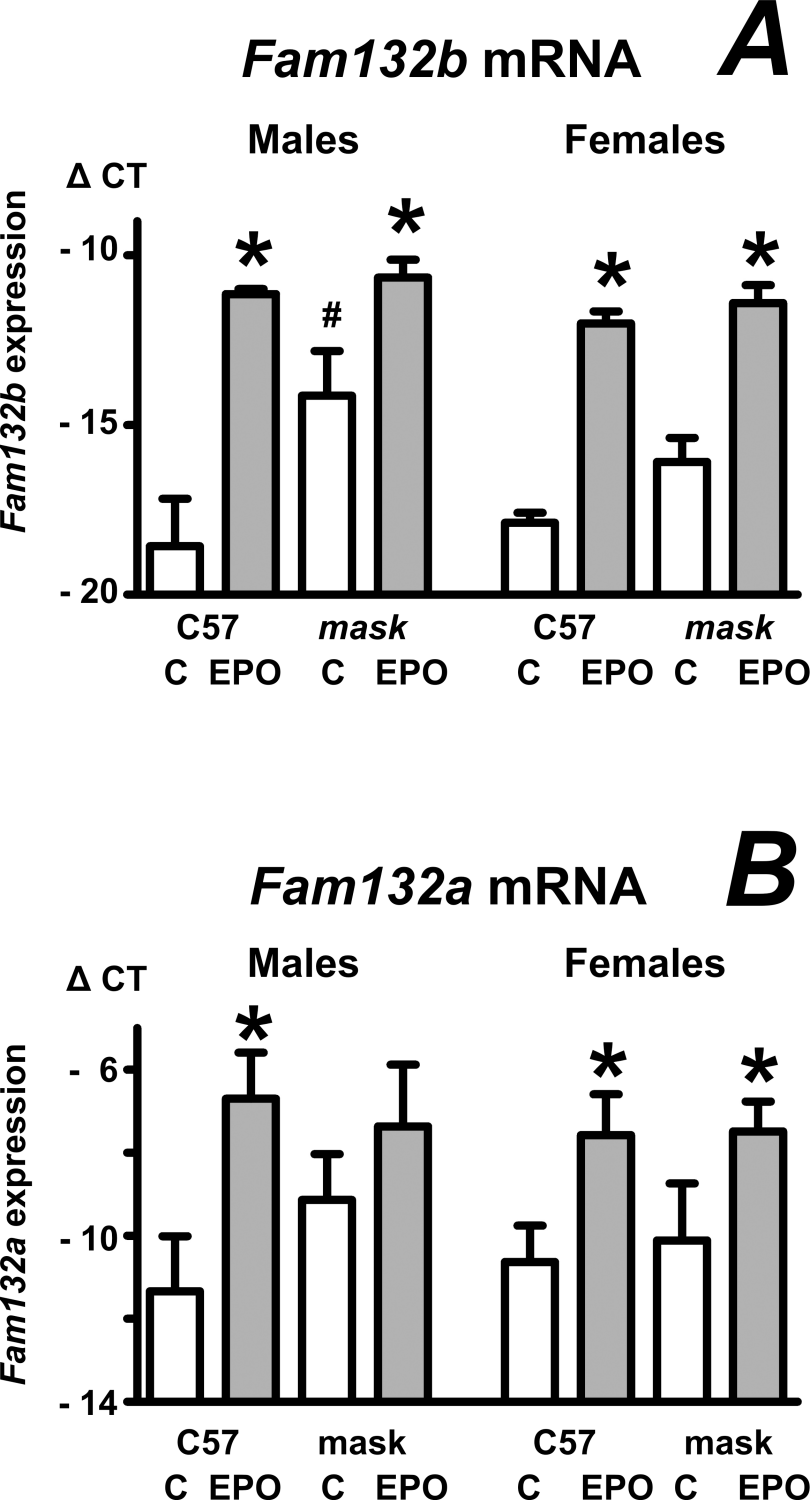
EPO increases *Fam132b* and *Fam132a* expression in the spleen of C57BL/6 and *mask* mice. A and B: Effect of EPO administration (50 IU/mouse daily for four days) on spleen *Fam132b* and *Fam132a* mRNA content in C57BL/6 (C57) and *mask* mice. Data are expressed as mean ± SD of Δ CT values obtained by real-time PCR (Δ CT = CT *Actb*–CT target), n = 5. Asterisks denote statistically significant difference between PBS-treated (C) groups and EPO-treated groups; hash sign denotes statistically significant difference between C57BL/6 mice and *mask* mice (p<0.05).

ERFE, encoded by the *Fam132b* gene, has been identified as the protein responsible for *Hamp* gene downregulation by erythropoietic activity [15]. It has recently been shown that *Fam132b* mRNA is upregulated by EPO in bone marrow of mice with a targeted disruption of *Tmprss6* gene [20]. Data presented in Fig 2A show that EPO also increases *Fam132b* mRNA content in the spleen of *mask* mice. In addition to *Fam132b* expression, splenic expression of *Fam132a* gene was increased by EPO treatment as well (Fig 2B). In agreement with data previously reported for *Fam132b* expression in the bone marrow [20] and spleen [32] of *Tmprss6*-/- mice, *Fam132b* mRNA content in the spleen of PBS-treated *mask* mice was higher than in PBS-treated C57BL/6 mice; for males, this difference reached statistical significance (Fig 2A).

### ERFE protein content is increased by EPO in both C57BL/6 and *mask* mice

**Fig 3:**
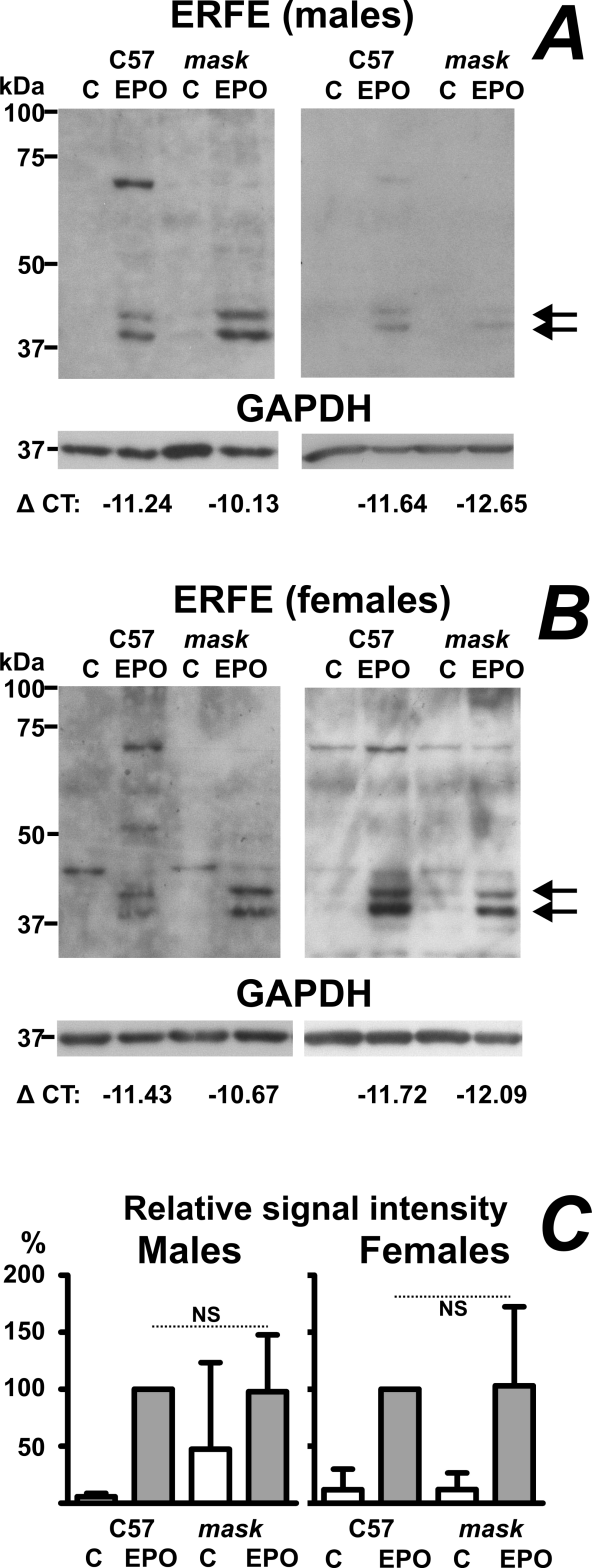
EPO increases ERFE protein synthesis in the spleen of C57BL/6 and *mask* mice. A: Two immunoblots of ERFE protein in spleen microsomes prepared from PBS-treated (C) and EPO-treated (50 IU/mouse daily for four days) male C57BL/6 (C57) and *mask* mice. Arrows denote the ERFE double band at approximately 39 and 42 kDa. GAPDH is used as loading control. Actual ΔCT values for *Fam132b* expression in analyzed samples are included for EPO-treated groups. B: Two immunoblots of ERFE protein in spleen microsomes prepared from female mice, experimental conditions as in panel A. C: Relative quantification of ERFE protein. GAPDH-normalized signals from PBS-treated C57BL/6 mouse, PBS-treated *mask* mouse and EPO-treated *mask* mouse were expressed as percentages of GAPDH-normalized signal from EPO-treated C57BL/6 mouse from the same blot. Data from individual blots were subsequently analyzed by 1-way ANOVA, n = 5. There was no significant difference between EPO-treated C57BL/6 mice and EPO-treated *mask* mice (NS: p ˃ 0.05).

At present, there are no data on the expression of ERFE protein in *Tmprss6*-mutated mice, with the exception of plasma ERFE levels determined by enzyme-linked immunosorbent assay [28]. We have recently reported the use of commercially available SC-246567 anti-myonectin antibody for ERFE determination in rats [30]; the same antibody also detects ERFE protein in mice (S1 Fig). The ERFE signal could be detected in both whole homogenates and microsomes prepared from spleens of EPO-treated C57BL/6 and EPO-treated *mask* mice (S2 Fig); however, in whole spleen homogenate the antibody detected relatively strong non-specific bands, whereas the use of microsomes diminished non-specific signals and enhanced ERFE detection. Therefore, the microsomal fraction was used in all subseqent studies on ERFE protein expression. In both whole homogenates and microsomes, ERFE is detected as a double band indicating different glycosylation pattern; treatment with PNGase F resulted in the detection of one single band at approximately 35 kDa [30]. For quantification by densitometry, only the lower band was used. In agreement with the low content of *Fam132b* mRNA in PBS-treated C57BL/6 mice (Fig 2A), ERFE protein was not detected in these animals (Fig 3A and 3B); administration of EPO resulted in ERFE protein detection in both C57BL/6 and *mask* mice. Comparison of individual blots (included as S3 Fig, S4 Fig and S2 Table) indicated no statistically significant difference in EPO-induced ERFE protein content between C57BL/6 mice and *mask* mice. Comparison between ERFE signal intensities and Δ CT values for *Fam132b* expression indicated significant linear correlation (S5 Fig), suggesting that ERFE protein synthesis is regulated mainly at the transcriptional level.

### Splenic transferrin receptor 2 protein is increased by EPO in both C57BL/6 and *mask* mice

**Fig 4:**
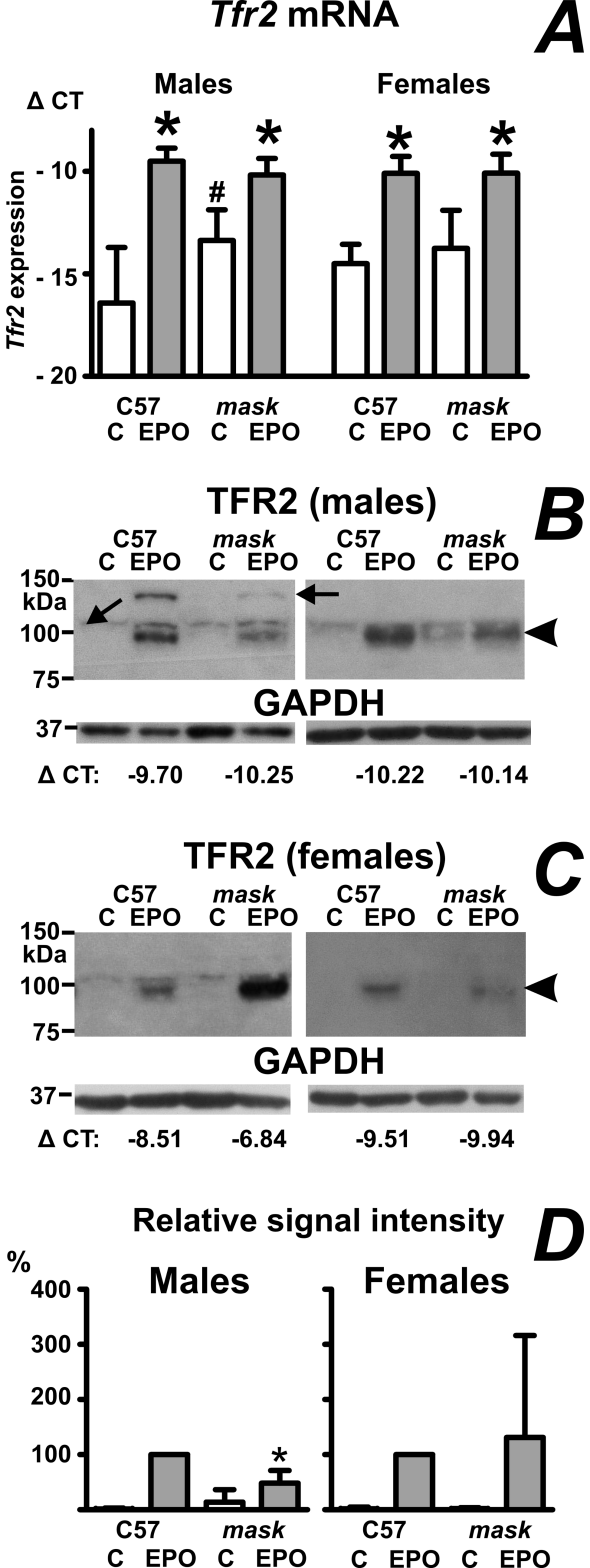
EPO increases TFR2 protein synthesis in the spleen of C57BL/6 and *mask* mice. A: Effect of EPO administration on splenic *Tfr2* mRNA content. Data are expressed as mean ± SD of Δ CT values obtained by real-time PCR (Δ CT = CT *Actb*–CT target), n = 5. Asterisk denotes statistically significant difference between PBS-treated (C) group and EPO-treated group, hash sign denotes statistically significant difference between C57BL/6 group and *mask* group (p<0.05). B: Two immunoblots of TFR2 protein in spleen microsomes prepared from male PBS-treated (C) and EPO-treated (50 IU/mouse daily for four days) C57BL/6 (C57) and *mask* mice. Arrowhead denotes the TFR2 protein band; arrows indicate bands which are non-specific. GAPDH is used as loading control. Actual ΔCT values for *Tfr2* expression in analyzed samples are included for EPO-treated groups. C: Two immunoblots of TFR2 protein in spleen microsomes prepared from female mice, experimental conditions as in panel B. D: Relative quantification of TFR2 protein. GAPDH-normalized signals from PBS-treated C57BL/6 mouse, PBS-treated *mask* mouse and EPO-treated *mask* mouse were expressed as percentages of signal from EPO-treated C57BL/6 mouse on the same blot. Data from individual blots were subsequently analyzed by 1-way ANOVA, n = 4. Asterisks denotes statistical significance between EPO-treated C57BL/6 mice and EPO-treated *mask* mice.

Several recent reports have highlighted the role of transferrin receptor 2 (TFR2) in erythropoiesis [32–34]. Since functional erythropoiesis is compromised in *mask* mice, it was of interest to compare the expression of splenic *Tfr2* mRNA between C57BL/6 and *mask* mice. As can be seen in Fig 4A, *Tfr2* mRNA was significantly induced by EPO treatment in both mouse strains. To verify whether the EPO-induced increase of *Tfr2* mRNA is mirrored by a detectable increase of TFR2 protein, we attempted to detect TFR2 in whole spleen homogenates and in spleen microsomes. In homogenates, we were not able to detect TFR2-related bands; however, a band corresponding to TFR2 protein was detected in microsomes prepared from EPO-treated C57BL/6 mice. Following PNGase F treatment, the band was shifted by about 6 kDa, confirming glycosylation of the detected protein (S6 Fig). Similarly to ERFE, TFR2 signal could not be detected in C57BL/6 mice treated with PBS; following EPO-treatment, the TFR2 band appeared in both C57BL/6 and *mask* mice (Fig 4B and 4C). Comparison of individual blots (included as S3 Fig, S4 Fig and S2 Table) indicated no statistically significant difference in EPO-induced TFR2 protein content between female C57BL/6 mice and *mask* mice; in male EPO-treated *mask* mice, the TFR2 protein content was lower than in male EPO-treated C57BL/6 mice (Fig 4D).

### Splenic ferroportin protein is decreased in EPO-treated *mask* mice

**Fig 5:**
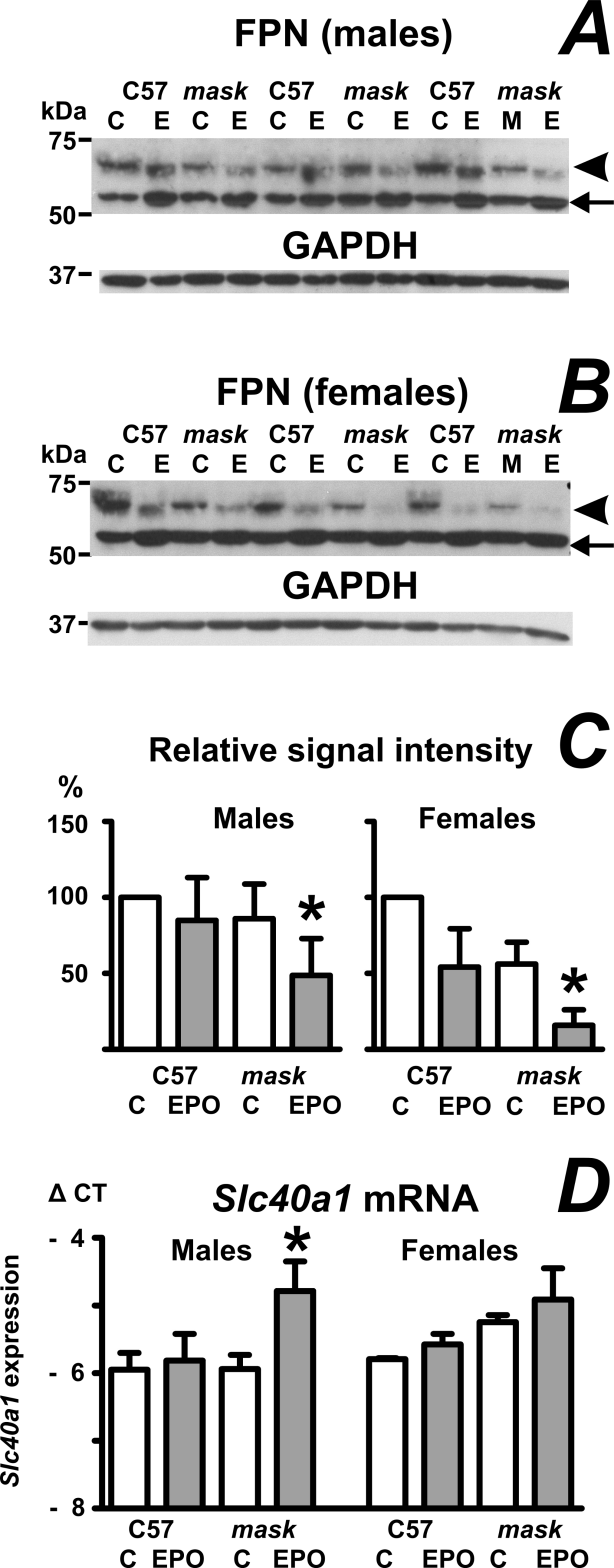
Effect of EPO administration on ferroportin protein content in the spleen. A and B: Ferroportin (FPN) protein content in spleen homogenates from PBS-treated (C) and EPO-treated (50 IU/mouse daily for four days) C57BL/6 (C57) and *mask* mice. Arrowhead denotes the ferroportin protein band; arrow indicates a non-specific band at 52 kDa. GAPDH is used as loading control. C: Relative quantification of FPN protein. Signals from EPO-treated C57BL/6 mouse, PBS-treated *mask* mouse and EPO-treated *mask* mouse were expressed as percentages of signal from PBS-treated C57BL/6 mouse on the same blot. Data from individual blots were subsequently analyzed by 1-way ANOVA, n = 6 for males and n = 5 for females. Asterisks denote statistical significance between EPO-treated C57BL/6 mice and EPO-treated *mask* mice. C = PBS-treated; E = EPO-treated groups. D: Effect of EPO administration on splenic *Slc40a1* mRNA content. Data are expressed as mean ± SD of Δ CT values obtained by real-time PCR (Δ CT = CT *Actb*–CT target), n = 3. Asterisk denotes statistically significant difference between PBS-treated (C) group and EPO-treated group (p<0.05).

Because *Hamp* gene expression is inappropriately high in *mask* mice [3], it was expected that splenic FPN will be inappropriately low, thus limiting macrophage iron export and compromising erythropoiesis. As noted previously by other groups [35,36], FPN detection by immunoblot requires modification of the standard electrophoresis protocol by omission of the usual denaturation step at 90°C (S7 Fig); following short incubation at 50°C, FPN was detected in spleen homogenates as a band of approximately 64 kDa (Fig 5A and 5B), which is in agreement with the predicted molecular mass of mouse FPN (62.7 kDa, Uniprot entry Q9JHI9). In addition to this band, the immunoblots also displayed a relatively strong band at approximately 52 kDa, which is regarded as a non-specific. Data in Fig 5A, 5B and 5C indicate that FPN protein is decreased in EPO-treated *mask* mice when compared to EPO-treated C57BL/6 mice (data analysis is included as S8 Fig and S3 Table). Interestingly, despite the markedly lowered *Hamp* gene expression in EPO-treated C57BL/6 mice, FPN protein in the spleen of these mice did not increase, and in some cases was even lower than in PBS-treated mice. This observation is in agreement with published results [35], and can be explained by EPO-induced increase in the number of erythroid cells present in the spleen [35], as also confirmed by flow cytometry analysis (S4 Table). Since splenic FPN is expressed mainly in macrophages [36], the EPO-induced increase erythroid cells decreases the amount of FPN per gram of spleen or per mg of total spleen protein, resulting in sometimes paradoxically lower FPN content in EPO-treated animals than in PBS-treated controls. The observed changes in FPN protein content were not related to changes in transcription, as *Slc40a1* mRNA tended to increase, rather than decrease, in EPO-treated *mask* mice (Fig 5D).

### Lack of TMPRSS6 proteolytic activity results in alternative cleavage of hemojuvelin *in vivo*

**Fig 6:**
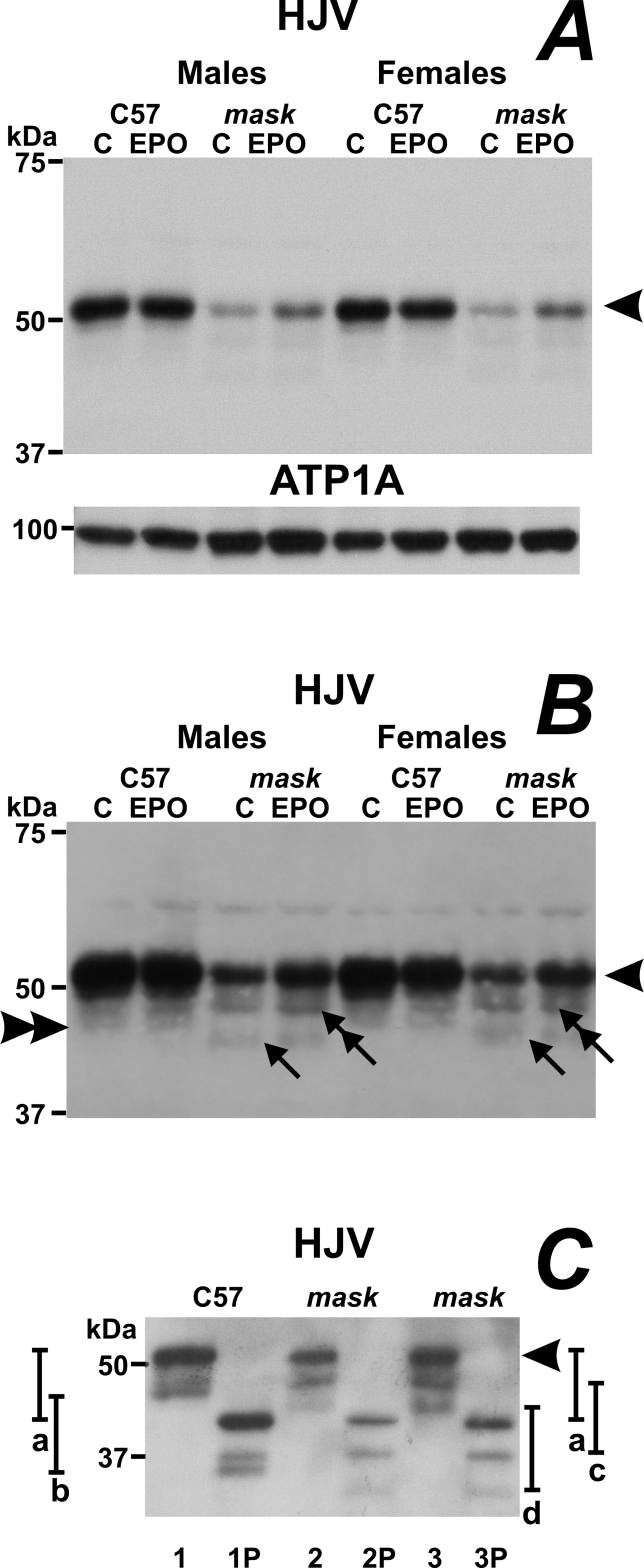
Absence of TMPRSS6-related proteolytic activity results in alternative cleavage of HJV protein. A: HJV protein content in plasma membrane-enriched fraction of mouse liver homogenates obtained from male and female C57BL/6 (C57) and *mask* mice treated with PBS (C) or EPO (50 IU/mouse daily for four days). Blot (10 min exposure) shows the full-length HJV protein band at approximately 52 kDa (arrowhead). ATP1A is used as loading control. B: Same blot as in Fig 5A exposed for 1 hour. Double arrowhead on left side of the blot denotes a minor cleaved band at approximately 47 kDa, which is absent from *mask* mice samples, arrow indicates a weak protein band of approximately 45 kDa present in only in *mask* mice; double arrow indicates a stronger minor band of approximately 48 kDa present in *mask* mice samples. C: Effect of PNGase F deglycosylation on HJV protein bands. Liver sample from C57BL/6 mouse (lane 1) and two *mask* mice (lane 2 and 3) before (lanes 1, 2 and 3) and after (lanes 1P, 2P and 3P) treatment with PNGase F. Arrowhead represents the main 52 kDa band. Line a indicates the PNGase-related shift of the main 52 kDa band; line b the shift of the C57BL/6-specific 47 kDa band; lines c a d indicate the shifts of the minor 48 and 45 kDa bands seen in *mask* mice. The PNGase-related shifts represent approximately 10 kDa for all four bands.

We have previously shown that liver HJV protein content is decreased in microsomes prepared from the livers of *mask* mice [26]; this observation was confirmed in the present study, which utilizes the plasma membrane-enriched 3000 g fraction (Fig 6A). By close examination of the HJV-specific bands in *mask* mice (Fig 6B), it is evident that the lack of TMPRSS6 proteolytic activity results not only in decreased HJV protein content, but also in alternative cleavage of the full-length HJV protein. In C57BL/6 mice, HJV is detected in the plasma membrane-enriched fraction as a major band of about 52 kDa, corresponding to the full-length protein (Fig 6A and 6B, arrowhead), and a weak minor band of about 47 kDa (Fig 6B, double arrowhead), which corresponds to a product cleaved at the N-terminus [29]. In *mask* mice, the cleaved 47 kDa band is not detected, but minor protein bands are apparent at about 45 kDa (Fig 6B, arrow) and 48 kDa (Fig 6B, double arrow). Treatment of the samples with PNGase F, which removes N-linked oligosaccharides, shows that the cleaved products retain all three oligosaccharide chains present in the full length protein (Fig 6C).

## Discussion

The main purpose of the study was to examine the effect of EPO administration on proteins participating in the regulation of iron metabolism. In particular, we were interested in the effect of EPO on the synthesis of ERFE in the spleen, since spleen is the main site of mouse stress erythropoiesis [37,38], and in the effect of *Tmprss6* mutation on HJV, since HJV is the proposed target of *Tmprss6*-related proteolytic activity [21].

Patients with *TMPRSS6* mutations display microcytic anemia which does not respond to EPO administration [17]. Initially, there has been some controversy as to whether *Tmprss6*-mutated mice are able to downregulate hepcidin expression and increase hemoglobin synthesis following EPO injection [18,19,39]. Results from this study confirm [19] the lack of response to EPO in *mask* mice, which lack the proteolytic domain of TMPRSS6 protein. Although *mask* mice administered EPO for four consecutive days display increased spleen size, their erythrocytes are small and neither hemoglobin level nor hematocrit are significantly increased by EPO treatment. Moreover, in agreement with *Tmprss6*-mutated mice [20], *mask* mice do not decrease *Hamp* mRNA following EPO treatment. The failure to downregulate *Hamp* expression is caused by hyperactivation of the phosphorylated SMAD signaling pathway [24,25] which controls *Hamp* expression in response to iron [40], as basal levels of *Id1* mRNA are higher in *mask* mice than in C57BL/6 mice, and are not decreased by EPO administration (Fig 1B).

The high expression of *Hamp* in *Tmprss6*-mutated mice decreases ferroportin protein content at the enterocyte basolateral membrane [4], resulting in decreased absorption of iron from the diet, depletion of iron stores and iron deficiency anemia. In addition to blocking iron uptake from the diet, high plasma hepcidin should also result in decreased ferroportin content in macrophages, and thus in slower recycling of iron from phagocytosed erythrocytes. In accordance with this concept, splenic FPN content in EPO-treated *mask* mice was decreased in comparison with EPO-treated C57BL/6 mice, suggesting that the decrease in iron recycling could contribute to the inability of EPO to at least partially normalize the low hemoglobin levels in *mask* mice, despite a functional response of spleen erythropoiesis to EPO documented by the significant increase in spleen size (Table 1).

The downregulation of *Hamp* by stress erythropoiesis is mediated by ERFE [16], a recently discovered erythroblast-derived protein encoded by *Fam132b* gene [15]. It has already been demonstrated that *Fam132b* mRNA increases following EPO administration in the bone marrow of *Tmprss6*-mutated mice [20]. Since stress erythropoiesis in rodents takes place mainly in the spleen [37], we now extend this information by determining *Fam132b* expression in the spleen of *mask* mice. Similarly to *Fam132b* expression in bone marrow [20], splenic *Fam132b* mRNA content increased to approximately similar extent both in C57BL/6 and *mask* mice. To examine whether the increase in *Fam132b* mRNA is also mirrored by an increase in ERFE protein synthesis, we examined ERFE protein content in the spleen of EPO-treated C57BL/6 and *mask* mice using a commercial anti-myonectin antibody. Data presented in Fig 3 show that the synthesis of ERFE protein is intact in spleens of *mask* mice, thus demonstrating that the inability to downregulate *Hamp* expression is caused by a defect downstream of ERFE protein synthesis. There was no significant difference between the amount of ERFE between EPO-treated C57BL/6 and EPO-treated mask mice. ERFE could be detected both in homogenates of whole spleen and in spleen microsomes; the use of microsomes decreased non-specific bands and increased ERFE band intensity. As the microsomal fraction contains membranes from the endoplasmic reticulum, ERFE detected in this fraction probably represents the newly synthesized protein. During maturation process, ERFE is glycosylated, as evidenced by the two bands detected on immunoblots. There was no difference in the glycosylation pattern between wild-type and *mask* mice, again indicating functional synthesis of ERFE protein in EPO-treated *mask* mice. The amount of ERFE protein detected in microsomes correlated with splenic *Fam132b* mRNA content, suggesting that ERFE protein synthesis is regulated mainly at the transcriptional level.

Interestingly, in addition to *Fam132b* expression, EPO treatment also increased splenic expression of *Fam132a* and *Tfr2* genes. FAM132A protein (adipolin or CTRP12) is reported to form heteromeric complexes with ERFE *in vitro* [41], and could therefore theoretically modulate ERFE-dependent signaling; however, this role of CTRP12 remains so far speculative, as we were unable to confirm its increased levels by immunoblotting. TFR2 protein has recently been demonstrated to play a role not only in the liver, but also in erythroid precursors [32–34], where it might function as a modulator of EPO signaling [42]. In the case of TFR2, we were able to document the effect of EPO not only at the mRNA level, but, by analyzing splenic microsomes, also at the protein level. EPO treatment increased TFR2 protein both in C57BL/6 and *mask* mice, suggesting that the failure of EPO to correct the microcytic anemia in *mask* mice is not related to defective synthesis of this protein. In male EPO-treated *mask* mice, TFR2 protein content was lower than in EPO-treated C57BL/6 mice (Fig 4B and 4D). As there was no difference in *Tfr2* mRNA content (Fig 4A), the results could indicate posttranscriptional regulation of splenic TFR2 protein. In the liver, TFR2 expression is posttranscriptionally regulated by diferric transferrin [43]; in erythroid cells *in vitro*, TFR2 is cleaved from the cell surface in iron deficiency [44]. It is at present not known whether such regulation occurs in the spleen *in vivo*; in this respect, the detection of TFR2 in spleen microsomes from EPO-treated mice by a well-established antibody [43,45] could open new possibilities in the study of the extrahepatic function of this protein.

Based on *in vitro* experiments, it has been proposed that the major physiological function of TMPRSS6 is to proteolytically cleave and thus inactivate hepatocyte HJV [21], a crucial component of the SMAD-dependent pathway of hepcidin regulation. When examining HJV protein in *in vivo* experiments by immunoblotting, we have previously shown that liver homogenates and microsomes from *mask* mice paradoxically display decreased rather than increased amount of HJV protein [26], an observation confirmed in the present study. To gain further insight on HJV cleavage, we now also examined the pattern of minor HJV protein bands, which can be detected after longer X-ray film exposure of immunoblots containing the plasma membrane-enriched fractions [29]. In C57BL/6 mice we observed, in addition to the major full-length HJV protein band of approximately 52 kDa, a slight minor protein band of approximately 47 kDa, which probably results from cleavage of the full length protein. Intriguingly, in samples from *mask* mice this minor band was absent, and minor protein bands of approximately 45 and 48 kDa could be detected instead. A possible interpretation of these data is that TMPRSS6 partially cleaves the full-length HJV to the 47 kDa fragment and that, in the absence of TMPRSS6, hemojuvelin is subjected to cleavage by another protease. The disappearance of the 47 kDa band in *mask* mice apparently represents an *in vivo* confirmation of the proposed [21] cleavage of HJV by TMPRSS6, although it does not exclude the possibility that, in addition to HJV, TMPRSS6 could have other physiological substrates as well [46,47,48].

Treatment with PNGase F demonstrates that both the 47 kDa HJV fragment from C57BL/6 mice and the 45 and 48 kDa HJV fragments from *mask* mice retain the same number of attached oligosaccharides as the full length HJV protein, which indicates cleavage N-terminally of the HJV oligosaccharide chains. Since the predicted N-terminal glycosylation site of human HJV is reportedly located at asparagine 118 (UniProt entry Q6ZVN8), the presented *in vivo* data are not compatible with the proposed HJV cleavage site by TMPRSS6 at arginine 121 [49]. In this respect, it is also interesting to note that the N-terminus of HJV, which is evidently affected by the loss of TMPRSS6-related proteolytic activity (Fig 6B), is also important for BMP protein binding [50,51]. Overall, the intriguing cleavage of HJV in *Tmprss6*-mutated mask mice, as well as the very recent identification of other possible *in vitro* substrates for TMPRSS6 [48], seems to indicate that more *in vivo* studies are needed on the interaction of TMPRSS6 with HJV, as well as on the possible interaction of TMPRSS6 with other components of the bone morphogenetic protein signaling pathway.

In conclusion, the presented study confirms that administration of EPO for four days to *mask* mice does not decrease liver *Hamp* expression and, despite increased spleen size, does not result in increase of hemoglobin levels. Using immunoblotting, it demonstrates that EPO-induced ERFE protein synthesis is intact in *mask* mouse spleen, and that both C57BL/6 and *mask* mice upregulate splenic TFR2 protein synthesis following EPO administration. As to the proposed mechanism of TMPRSS6 mode of action, it supports the *in vivo* interaction between TMPRSS6 and HJV, and, intriguingly, demonstrates alternative cleavage of HJV in *mask* mouse livers.

## Supporting information

S1 Table Primers used for PCR analysis. (PDF)

S2 Table Data related to ERFE and TFR2 immunoblots in S3 and S4 Figs. (PDF)

S3 Table Data related to FPN immunoblots in Fig 5 and S8 Fig. (PDF)

S4 Table Flow cytometry analysis of spleen cells. (PDF)

S1 Fig Validation of the anti-ERFE antibody. (PDF)

S2 Fig Detection of ERFE in whole spleen homogenates and spleen microsomes. (PDF)

S3 Fig Immunoblotting of ERFE and TFR2 in spleen microsomes from male C57BL/6 and *mask* mice. (PDF)

S4 Fig Immunoblotting of ERFE and TFR2 in spleen microsomes from female C57BL/6 and *mask* mice. (PDF)

S5 Fig Correlation between ERFE signal intensity and *Fam132b* Mrna. (PDF)

S6 Fig Examination of the glycosylation pattern of splenic TFR2 protein. (PDF)

S7 Fig Negative effect of denaturation on ferroportin protein detection. (PDF)

S8 Fig Immunoblotting of FPN in spleen homogenates from C57BL/6 and *mask* mice. (PDF)
