# Supplementary material for: Effect of erythropoietin administration on proteins participating in iron homeostasis in Tmprss6-mutated mask mice
Source: PLoS One. 2017 Oct 26;12(10):e0186844. doi: 10.1371/journal.pone.0186844 (PMC5658091; doi:10.1371/journal.pone.0186844)
Supplement: S1 Table — (PDF) [file pone.0186844.s001.pdf]

S1 Table. Primers used for PCR analysis

Actb: GCTACAGCTTCACCACCACA and GGTCTTTACGGATGTCAACG,

Hamp: CTGAGCAGCACCACCTATCTC and TGGCTCTAGGCTATGTTTTGC,

Id1: CGAGGTGGTACTTGGTCTGTC and CTGCAGGTCCCTGATGTAGTC,

Slc40a1: ATCGGTCTTTGGTCCTTTGAT and ATTGCCACAAAGGAGACTGAA,

Fam132b: ATGCTGTTCGTCAAGCAGAGT and CCTTCAGCAGAACCTCAGATG,

Fam132a: CCTCTAGAAAACGGTGTCGTG and CAGTAAGGCCTCTGGGGTAAC,

Tfr2: CTGGGAACTGGAGACCCTTAC and AAGGAGAGCCTGAGAGGTGAC.

To confirm linear correlation between template cDNA and Δ CT, cDNA obtained from EPO-treated spleen was diluted from 2x to 4096x and Δ CT values obtained *Actb*, *Fam132b*, *Fam132a* and *Tfr2* primers were recorded. Amplification was linear over the range of the experiment.
