# Supplementary material for: Effect of erythropoietin administration on proteins participating in iron homeostasis in Tmprss6-mutated mask mice
Source: PLoS One. 2017 Oct 26;12(10):e0186844. doi: 10.1371/journal.pone.0186844 (PMC5658091; doi:10.1371/journal.pone.0186844)
Supplement: S2 Table — (PDF) [file pone.0186844.s002.pdf]

S2 Table. Data related to ERFE and TFR2 immunoblots shown in S3 and S4 Figs.

A) Male mice (S3 Fig)

S3 Fig A

Mouse:		C57	C57	*mask*	*Mask*
Treatment:		PBS	EPO	PBS	EPO
Spleen weight (mg):		68	241	77	178
Fam132b delta CT		-17,93	-10,15	-12,72	-9,86
Tfr2 delta CT		-16,29	-9,14	-12,59	-9,73
ERFE Signal:		10827,5	135882,5	5826,375	230645,2
TFR2 Signal:		9935,76	288177,5	6647,25	95981,75
GAPDH Signal:		213041,2	219585,4	381557,6	317525,2
Normalized ERFE		5,08235	61,88139	1,526997	72,63839
Normalized TFR2		4,663774	131,2371	1,742135	30,22807
Relative to	ERFE	**8,21**	**100**	**2,47**	**117,38**
C57EPO(%):	TFR2	**3,55**	**100**	**1,33**	**23,03**

S3 Fig B

Mouse:		C57	C57	*mask*	*Mask*
Treatment:		PBS	EPO	PBS	EPO
Spleen weight (mg):		32	164	146	212
Fam132b delta CT		-20,77	-10,96	-10,14	-10,27
Tfr2 delta CT		-20,03	-9,15	-11,25	-9,56
ERFE Signal:		3917,563	70523,75	113257,2	128793,7
TFR2 Signal:		2528,26	364427,2	146427,3	277558,9
GAPDH Signal:		573005,6	436299,6	436981,7	459815,3
Normalized ERFE		0,683687	16,16406	25,91806	28,00988
Normalized TFR2		0,445425	86,35375	34,4394	62,04718
Relative to	ERFE	**4,23**	**100**	**160,34**	**173,29**
C57EPO(%):	TFR2	**0,52**	**100**	**39,88**	**71,85**

S3 Fig C

Mouse:		C57	C57	*mask*	*Mask*
Treatment:		PBS	EPO	PBS	EPO
Spleen weight (mg):		87	250	84	241
Fam132b delta CT		-20,06	-10,73	ND	-10,58
Tfr2 delta CT		-12,83	-8,84	ND	-9,11
ERFE Signal:		756	140173,3	258	212531
TFR2 Signal:		926	80062	565	59558
GAPDH Signal:		400730	350388,4	422672	367830,2
Normalized ERFE		0,188656	40,00512	0,06104	57,77965
Normalized TFR2		0,231078	22,8495	0,133673	16,19171
Relative to	ERFE	**0,47**	**100**	**0,15**	**144,43**
C57EPO(%):	TFR2	**1,01**	**100**	**0,59**	**70,86**

S3 Fig D

Mouse:		C57	C57	*mask*	*Mask*
Treatment:		PBS	EPO	PBS	EPO
Spleen weight (mg):		66	209	82	160
Fam132b delta CT		-18,73	-11,64	-18,17	-12,65
Tfr2 delta CT		ND	ND	ND	ND
ERFE Signal:		2371,484	60737,06	2302,25	35944,38
TFR2 Signal:		ND	ND	ND	ND
GAPDH Signal:		540685	268544	357122	363607
Normalized ERFE		0,438607	22,61717	0,644668	9,885501
Relative to	ERFE	**1,94**	**100**	**2,85**	**43,71**
C57EPO(%):	TFR2	ND	ND	ND	ND

S3 Fig E

Mouse:		C57	*Mask*
Treatment:		EPO	EPO
Spleen weight (mg):		213	133
Fam132b delta CT		ND	ND
Tfr2 delta CT		ND	ND
ERFE Signal:		24,06527	20,32269
TFR2 Signal:		61,5021	33,80647
GAPDH Signal:		543818,8	476502,2
Normalized ERFE		24,06527	20,32269
Normalized TFR2		0,011309	0,007095
Relative to	ERFE	**100**	**84,49**
C57EPO(%):	TFR2	**100**	**62,73**

Summarized:	C57 PBS	C57 EPO	mask	mask EPO
Relative ERFE	**8,21**	**100**	**2,47**	**117,38**
	**4,23**	**100**	**160,34**	**173,29**
	**0,47**	**100**	**0,15**	**144,43**
	**1,94**	**100**	**2,85**	**43,71**
		**100**		**84,49**
Relative TFR2	**3,55**	**100**	**1,33**	**23,03**
	**0,52**	**100**	**39,88**	**71,85**
	**1,01**	**100**	**0,59**	**70,86**
		**100**		**62,73**

B) Female mice (S4 Fig)

S4 Fig A

Mouse:		C57	C57	mask	Mask
Treatment:		PBS	EPO	PBS	EPO
Spleen weight (mg):		60	185	85	170
Fam132b delta CT		18,72	12,88	16,07	9,93
Tfr2 delta CT		16,04	10,99	15,33	9,17
ERFE Signal:		15442	41363	16509,33	67142
TFR2 Signal:		500	172664	4676,9	626627,8
GAPDH Signal:		641819,6	563275,8	696073,4	501945
Normalized ERFE		2,405972	7,343294	2,371781	13,37637
Normalized TFR2		0,077904	30,65354	0,671898	124,8399
Relative to	ERFE	**32,76**	**100**	**32,30**	**182,16**
C57EPO(%)	TFR2	**0,25**	**100**	**2,19**	**407,26**

S4 Fig B

Mouse:		C57	C57	mask	Mask
Treatment:		PBS	EPO	PBS	EPO
Spleen weight (mg):		68	308	64	140
Fam132b delta CT		17,83	11,43	18,66	10,67
Tfr2 delta CT		14,73	9,51	16,11	9,94
ERFE Signal:		534	69684,98	754	147038,5
TFR2 Signal:		6501	100404	607	65232
GAPDH Signal:		389420,6	322280	375791,2	416859
Normalized ERFE		0,137127	21,6225	0,200643	35,27297
Normalized TFR2		1,669403	31,15428	0,161526	15,64846
Relative to	ERFE	**0,63**	**100**	**0,93**	**163,13**
C57EPO(%)	TFR2	**5,36**	**100**	**0,52**	**50,23**

S4 Fig C

Mouse:		C57	C57	mask	Mask
Treatment:		PBS	EPO	PBS	EPO
Spleen weight (mg):		85	175	73	160
Fam132b delta CT		18,32	11,72	14,48	12,09
Tfr2 delta CT		14,07	9,89	12,04	10,15
ERFE Signal:		316	306230	67815	331361
GAPDH Signal:		637936,6	244239	446071	386690
TFR2 Signal:		1213	70192	6707,333	20402,5
GAPDH Signal:		637936,6	244239	446071	386690
Normalized ERFE		2,39108	98,77447	12,79294	81,82905
Normalized TFR2		0,026033	21,60683	0,866605	3,060751
Relative to	ERFE	**2,42**	**100**	**12,95**	**82,84**
C57EPO(%)	TFR2	**0,12**	**100**	**4,01**	**14,17**

S4 Fig D

Mouse:		C57	Mask
Treatment:		EPO	EPO
Spleen weight (mg):		183	160
Fam132b delta CT		ND	ND
Tfr2 delta CT		ND	ND
ERFE Signal:		142333,5	134536,2
TFR2 Signal:		394849,1	264545
GAPDH Signal:		607964,2	777323,6
Normalized ERFE		23,41149	17,30762
Normalized TFR2		64,94611	34,0328
Relative to	ERFE	**100**	**73,93**
C57EPO(%)	TFR2	**100**	**52,40**

S4 Fig E

Mouse:		C57	mask	Mask
Treatment:		EPO	PBS	EPO
Spleen weight (mg):		388	64	100
Fam132b delta CT		11,19	18,5	13,02
ERFE Signal:		147431,8	9786,108	44179,24
GAPDH Signal:		212156	424134	406810
Normalized ERFE		69,49	2,31	10,86
Relative to C57EPO(%)	ERFE	**100**	**3,32**	**15,63**

Summarized:	C57 PBS	C57 EPO	mask	mask EPO
Relative ERFE	**32,76**	**100**	**32,30**	**182,16**
	**0,63**	**100**	**0,93**	**163,13**
	**2,42**	**100**	**12,95**	**82,84**
		**100**		**73,93**
		**100**	**3,32**	**15,63**
Relative TFR2	**0,25**	**100**	**2,19**	**407,26**
	**5,36**	**100**	**0,52**	**50,23**
	**0,12**	**100**	**4,01**	**14,1**
		**100**		**52,40**

ERFE, TFR2 and GAPDH signals were obtained from blots shown in S3 Fig (males) and S4 Fig (females) by densitometric analysis using Image Studio Lite from LI-COR Biosciences. ERFE and TFR2 signals were normalized to GAPDH signals and expressed as a percentage of GAPDH-normalized signal from EPO-treated C57BL/6 mice (bold font). Summarized values from individual blots were analyzed by one-way ANOVA followed by Tukey post test. Results are graphed in Fig 3C and Fig 4D.
