# Supplementary material for: Effect of erythropoietin administration on proteins participating in iron homeostasis in Tmprss6-mutated mask mice
Source: PLoS One. 2017 Oct 26;12(10):e0186844. doi: 10.1371/journal.pone.0186844 (PMC5658091; doi:10.1371/journal.pone.0186844)
Supplement: S3 Table — (PDF) [file pone.0186844.s003.pdf]

S3 Table. Data related to ferroportin immunoblots shown in Fig 5 and S8 Fig.

FPN males:

Fig 5 A

**Mouse:**	C57	C57	mask	mask	C57	C57	mask	mask
**Treatment:**	PBS	EPO	PBS	EPO	PBS	EPO	PBS	EPO
FPN Signal	399707,5	314560,5	256498,5	208065	203925	128668	230207	143673
GAPDH Signal:	586593,1	419091,3	576482,1	505624,7	493866,2	464729,9	468861,7	462951,8
**Normalized FPN**	68,1405	75,05776	44,49375	41,15008	41,29155	27,68662	49,09912	31,03412
**Relative values:**	**100**	**110,15**	**65,29**	**60,39**	**100**	**67,05**	**118,90**	**75,15**

**Mouse:**	C57	C57	mask	mask
**Treatment:**	PBS	EPO	PBS	EPO
FPN Signal	287350	215317	241836	154531
GAPDH Signal:	495769,6	372524,3	581990,5	447150,2
**Normalized FPN**	57,9604	57,79945	41,55325	34,55908
**Relative values:**	**100**	**99,72**	**71,69**	**59,62**

S8 Fig

**Mouse:**	C57	C57	mask	mask	C57	C57	mask	mask
**Treatment:**	PBS	EPO	PBS	EPO	PBS	EPO	PBS	EPO
FPN Signal	281856	240096	253502	189854	257994,3	196791,2	200390,9	63202,6
GAPDH Signal:	340148	275904	360492	388824	610442	510891	437331	572624
**Normalized FPN**	82,86275	87,02157	70,32112	48,82775	42,26353	38,51922	45,82134	11,03736
**Relative values:**	**100**	**105,01**	**84,86**	**58,92**	**100**	**91,14**	**108,41**	**26,11**

**Mouse:**	C57	C57	mask	mask
**Treatment:**	PBS	EPO	PBS	EPO
FPN Signal	232592,1	75120,2	151614,6	25719,28
GAPDH Signal:	631180,1	557836,6	614663,4	588450
**Normalized FPN**	36,85036	13,46635	24,66628	4,370683
**Relative values:**	**100**	**36,54**	**66,93**	**11,86**

FPN females:

Fig 5 B

**Mouse:**	C57	C57	mask	mask	C57	C57	mask	mask
**Treatment:**	PBS	EPO	PBS	EPO	PBS	EPO	PBS	EPO
FPN Signal	589869,1	182737,7	252493,7	102640,6	343366,1	114897,9	162481,2	13849,88
GAPDH Signal:	477811,8	380786	477619,5	427331,7	437326,3	419204,3	512042,7	391671,3
**Normalized FPN**	123,4522	47,9896	52,86503	24,01894	78,51485	27,40856	31,73196	3,536096
**Relative values:**	**100**	**38,87**	**42,82**	**19,45**	**100**	**34,90**	**40,41**	**4,50**

Mouse:	C57	C57	mask	mask
**Treatment:**	PBS	EPO	PBS	EPO
FPN Signal	323251,1	57473,39	124824,6	37659,02
GAPDH Signal:	443724	358721	385451	446300
**Normalized FPN**	72,84959	16,02175	32,38403	8,438051
**Relative values:**	**100**	**21,99**	**44,45**	**11,58**

S8 Fig

Mouse:	C57	C57	mask	mask	C57	C57	mask	mask
**Treatment:**	PBS	EPO	PBS	EPO	PBS	EPO	PBS	EPO
FPN Signal	781504,9	594656	600917,1	124821,6	754849,3	457701,8	475811,7	106361,6
GAPDH Signal:	622841,6	609518,7	637934,8	590522,6	595543,3	620408,5	666522,4	590250,2
**Normalized FPN**	125	98	94	21	127	74	71	18
**Relative values:**	**100**	**77,75**	**75,07**	**16,84**	**100**	**58,20**	**56,32**	**14,21**

Relative values males:

C57 PBS	C57 EPO	mask PBS	mask EPO
**100**	**110,15**	**65,29**	**60,39**
**100**	**110,15**	**118,90**	**75,15**
**100**	**99,72**	**71,69**	**59,62**
**100**	**105,01**	**84,86**	**58,92**
**100**	**91,14**	**108,41**	**26,11**
**100**	**36,54**	**66,93**	**11,86**

Relative values females:

C57 PBS	C57 EPO	mask PBS	mask EPO
**100**	**38,87**	**42,82**	**19,45**
**100**	**34,90**	**40,41**	**4,50**
**100**	**21,99**	**44,45**	**11,58**
**100**	**77,75**	**75,07**	**16,84**
**100**	**58,20**	**56,32**	**14,21**

FPN and GAPDH signals were obtained from blots shown in Fig 5 and S8 Fig by densitometric analysis using Image Studio Lite from LI-COR Biosciences.
FPN signals were normalized to GAPDH signals and expressed as a percentage of GAPDH-normalized FPN signal from PBS-treated C57BL/6 mice (bold font). Summarized values from individual blots were analyzed by one-way ANOVA followed by Tukey post test. Results are graphed in Fig 5 C.
