# Supplementary material for: Effect of erythropoietin administration on proteins participating in iron homeostasis in Tmprss6-mutated mask mice
Source: PLoS One. 2017 Oct 26;12(10):e0186844. doi: 10.1371/journal.pone.0186844 (PMC5658091; doi:10.1371/journal.pone.0186844)
Supplement: S4 Table — (PDF) [file pone.0186844.s004.pdf]

S4 Table: Flow cytometry analysis of spleen cells.

Males:

**C57**				**C57EPO**				**mask**				**mask EPO**			
Spleen (mg)	GM +	B220 +	**Ter119 +**	Spleen (mg)	GM +	B220 +	**Ter119 +**	Spleen (mg)	GM +	B220 +	**Ter119 +**	Spleen (mg)	GM +	B220 +	**Ter119 +**
32	7,99	63,66	28,34	164	8,91	36,03	55,06	77	10,99	72,24	16,76	178	5,27	36,6	58,13
68	7,71	61,59	30,68	241	4,44	25,1	70,45	146	7,32	40,18	52,5	212	4,2	24,11	71,7

Females:

**C57**				**C57EPO**				**mask**				**mask EPO**			
Spleen (mg)	GM +	B220 +	**Ter119 +**	Spleen (mg)	GM +	B220 +	**Ter119 +**	Spleen (mg)	GM +	B220 +	**Ter119 +**	Spleen (mg)	GM +	B220 +	**Ter119 +**
60	6,02	49,2	35,48	185	4,58	19,53	68,78	85	11,63	50,92	30,69	170	7,73	25,14	58,97
68	12,12	47,01	33,08	308	5,15	15,36	72,49	70	12,18	52,59	27,86	100	10,69	25,47	56,18

Two mice per each group were analyzed. Suspensions of spleen cells in PBS were prepared from pieces of spleen (10-20 mg) by a loose-fitting glass homogenizer. Approximately 3 x 10^6 cells were incubated at 4^oC in the dark with the following fluorescently labelled BioLegend antibodies: 1) a mixture of APC/Cy7 anti mouse Ly-6G/Ly-6C (a marker of granulocytes) antibody, clone RB6-8C5, and APC/Cy7 anti-mouse CD11b (a marker of granulocytes and monocytes/macrophages).antibody, clone M1/70; 2) BV605 anti mouse CD45R (a B-cell marker) antibody, clone RA3-6B2; 3) APC anti-mouse TER119 (a marker of erythroid precursor cells) antibody, clone TER119. After 20 min, the cells were washed, diluted and analyzed on FACSAria II flow cytometer (BD Biosciences). Table expresses the relative content (in %) of granulocytes/monocytes/macrophages (GM +), B-cells (B220 +) and erythrocyte precursors (Ter 119 +).

Data show that both C57BL/6 and *mask* mice increase the proportion of Ter119-positive cells following EPO treatment.
