# Supplementary material for: Effect of erythropoietin administration on proteins participating in iron homeostasis in Tmprss6-mutated mask mice
Source: PLoS One. 2017 Oct 26;12(10):e0186844. doi: 10.1371/journal.pone.0186844 (PMC5658091; doi:10.1371/journal.pone.0186844)
Supplement: S1 Fig — (PDF) [file pone.0186844.s005.pdf]

S1 Fig. Validation of the anti-ERFE antibody.

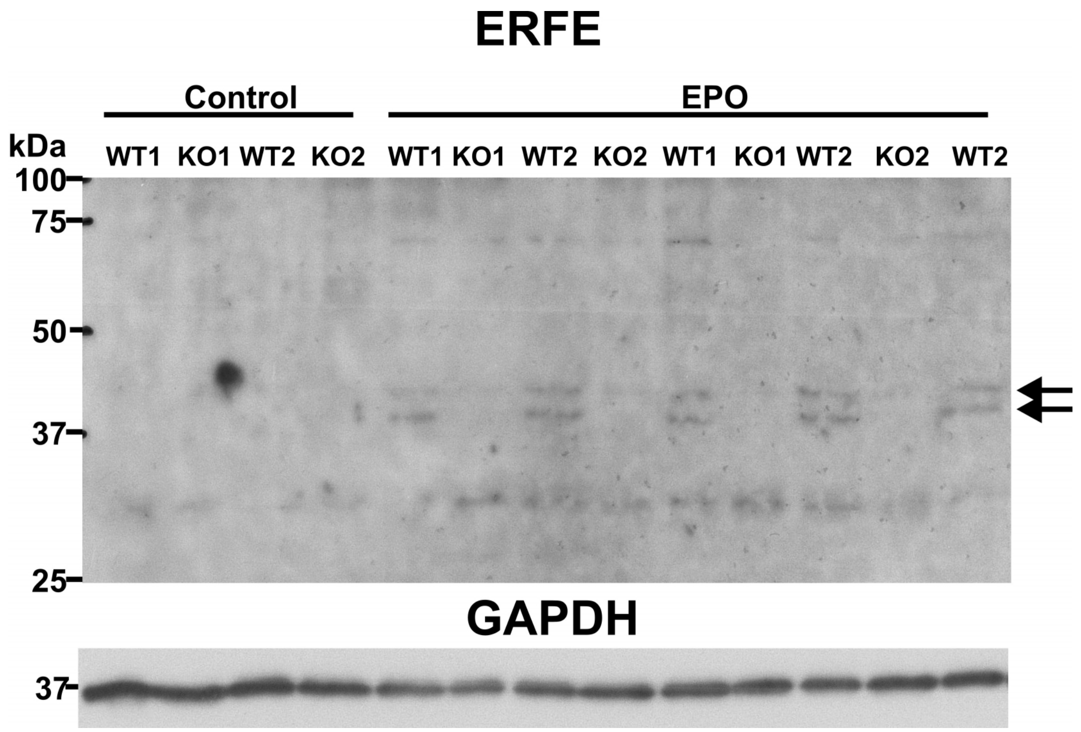

Microsomes were prepared from two sets of mouse spleens from 2 month old male *Fam132b*+/+ (WT) and *Fam132b*-/- (KO) control mice, or from *Fam132b*+/+ and *Fam132b*-/- mice treated with erythropoietin (EPO) 15 hours before sacrifice. Following standard immunoblotting procedure, ERFE was detected by SC-246567 anti-myonectin antibody at 1:200 dilution.

ERFE could not be detected in control mice. EPO treatment for 15 hours enabled ERFE detection (arrows) in *Fam132b*+/+ mice, but not in *Fam132b*-/- mice. It is therefore concluded that the two bands at approximately 42 and 39 kDa (arrows) represent ERFE protein.
