# Supplementary material for: Effect of erythropoietin administration on proteins participating in iron homeostasis in Tmprss6-mutated mask mice
Source: PLoS One. 2017 Oct 26;12(10):e0186844. doi: 10.1371/journal.pone.0186844 (PMC5658091; doi:10.1371/journal.pone.0186844)
Supplement: S2 Fig — (PDF) [file pone.0186844.s006.pdf]

S2 Fig. Detection of ERFE in whole spleen homogenates and spleen microsomes

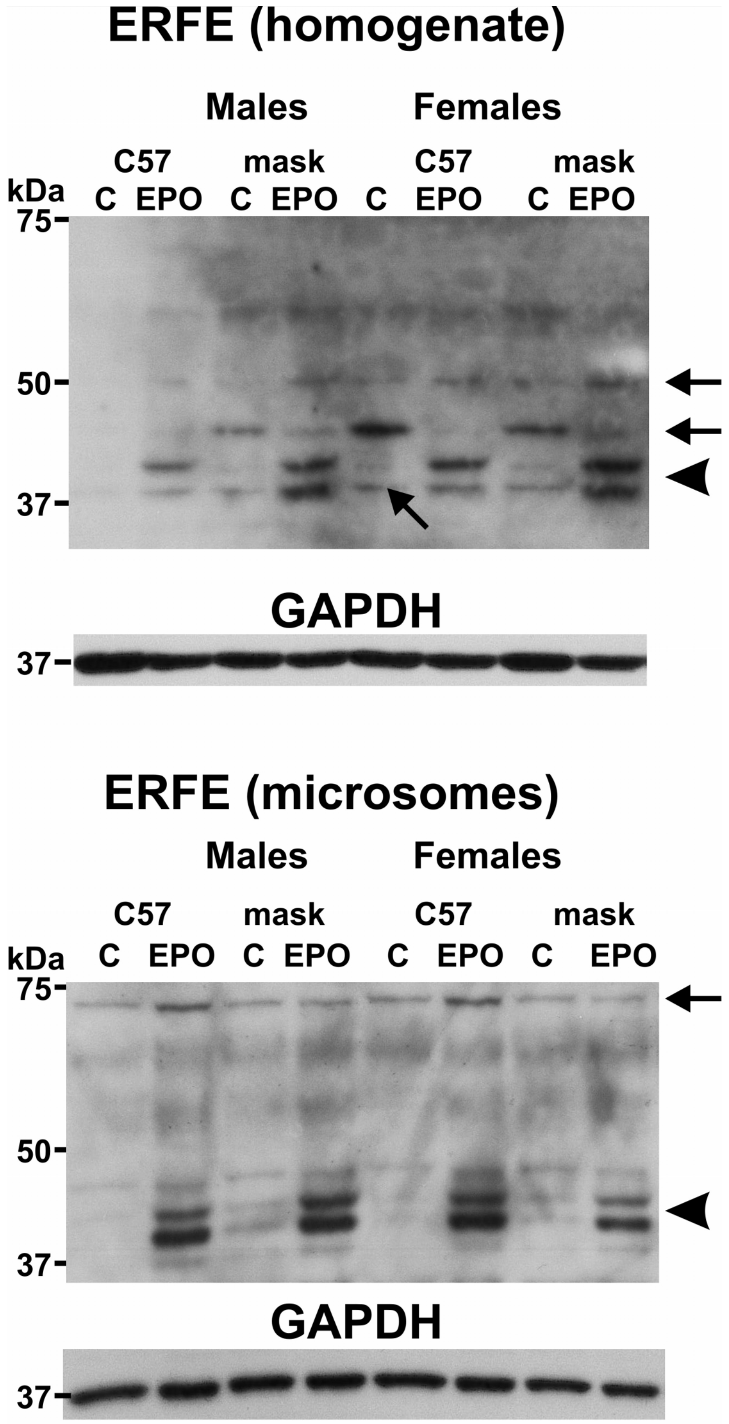

ERFE protein was detected by the SC-246567 antibody in spleen homogenates (prepared in 1% NP-40 buffer) and spleen microsomes. C57BL/6 (C57) and *mask* mice were treated with PBS (C) or EPO for four days. Arrowheads denote the ERFE-specific bands at approximately 39 and 42 kDa, arrows denote non-specific bands. GAPDH is used as loading control.
