# Supplementary material for: Effect of erythropoietin administration on proteins participating in iron homeostasis in Tmprss6-mutated mask mice
Source: PLoS One. 2017 Oct 26;12(10):e0186844. doi: 10.1371/journal.pone.0186844 (PMC5658091; doi:10.1371/journal.pone.0186844)
Supplement: S3 Fig — (PDF) [file pone.0186844.s007.pdf]

S3 Fig. Immunoblotting of ERFE and TFR2 in spleen microsomes from male C57BL/6 and *mask* mice

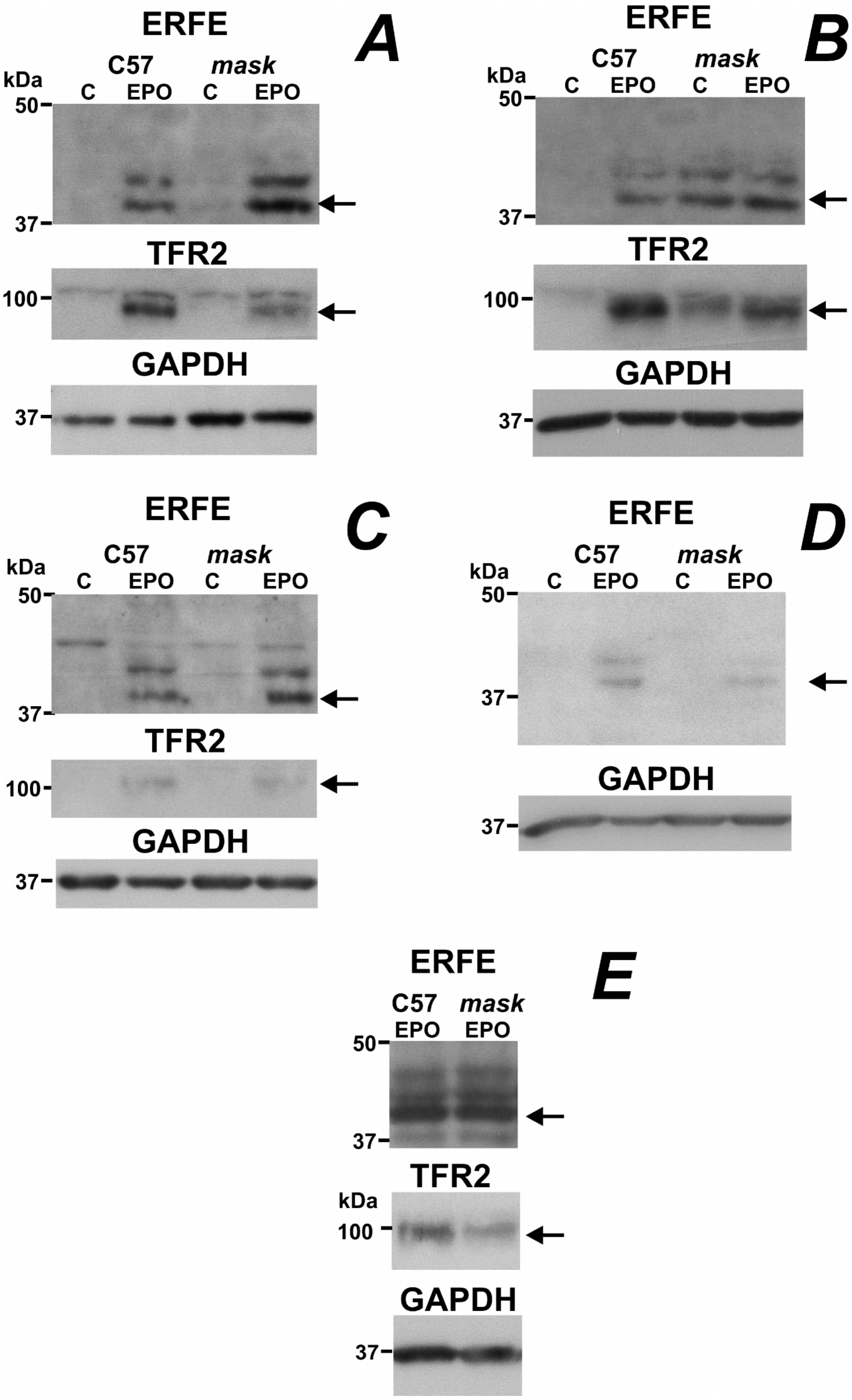

Panels A-E: Immunoblots of ERFE and TFR2 protein in spleen microsomes prepared from male PBS-treated (C) and EPO-treated (50 IU/mouse daily for four days) C57BL/6 (C57) and *mask* mice. Arrows denote the ERFE and TFR2 bands quantified in S2 Table.
