# Supplementary material for: Effect of erythropoietin administration on proteins participating in iron homeostasis in Tmprss6-mutated mask mice
Source: PLoS One. 2017 Oct 26;12(10):e0186844. doi: 10.1371/journal.pone.0186844 (PMC5658091; doi:10.1371/journal.pone.0186844)
Supplement: S5 Fig — (PDF) [file pone.0186844.s009.pdf]

S5 Fig. Correlation between ERFE signal intensity and *Fam132b* mRNA

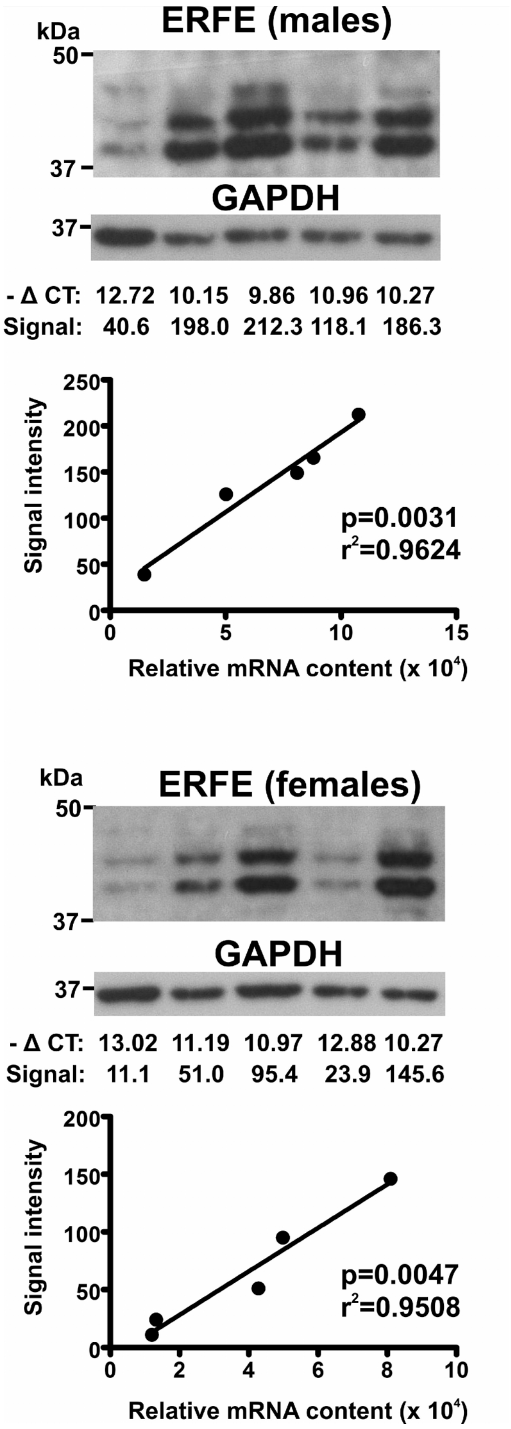

ERFE signal intensities from five samples (spleen microsomes from EPO-treated mice) were normalized to GAPDH controls; the resulting signals were plotted against relative mRNA content of the same samples calculated from the Δ CT values. The correlation was significant for both male and female mice.
