# Supplementary material for: Effect of erythropoietin administration on proteins participating in iron homeostasis in Tmprss6-mutated mask mice
Source: PLoS One. 2017 Oct 26;12(10):e0186844. doi: 10.1371/journal.pone.0186844 (PMC5658091; doi:10.1371/journal.pone.0186844)
Supplement: S6 Fig — (PDF) [file pone.0186844.s010.pdf]

S6 Fig. Examination of the glycosylation pattern of splenic TFR2 protein

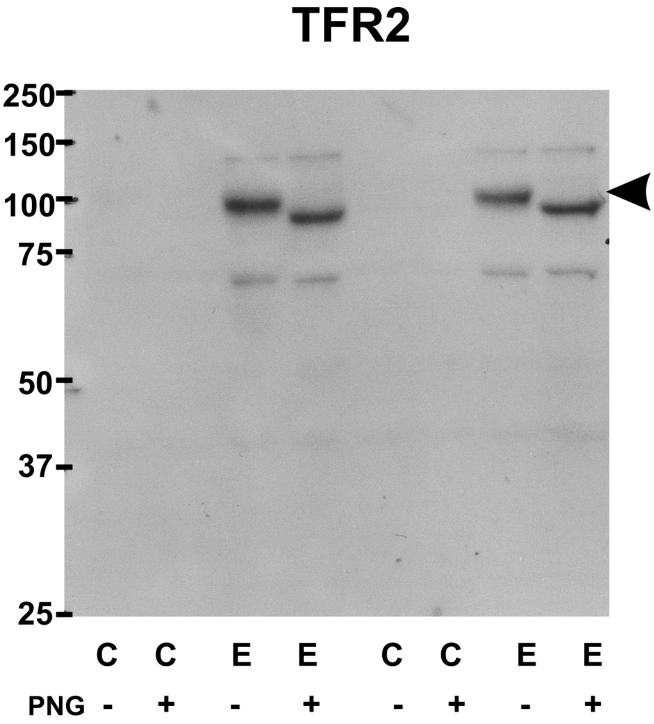

Spleen microsomes from control (C) or EPO-treated (E) male mice were subjected to treatment with PNGase F (PNG +), which removes N-linked oligosaccharides; water was added instead of PNGase F to control samples (PNG -). Samples were analyzed by immunoblotting with an established TFR2 antibody (TFR21 from Alpha Diagnostics International). Arrowhead denotes the glycosylated TFR2 band, which was shifted by about 6 kDa by PNGase F treatment, indicating that splenic TFR2 is glycosylated.
