# Supplementary material for: Effect of erythropoietin administration on proteins participating in iron homeostasis in Tmprss6-mutated mask mice
Source: PLoS One. 2017 Oct 26;12(10):e0186844. doi: 10.1371/journal.pone.0186844 (PMC5658091; doi:10.1371/journal.pone.0186844)
Supplement: S7 Fig — (PDF) [file pone.0186844.s011.pdf]

S7 Fig. Negative effect of denaturation on ferroportin protein detection

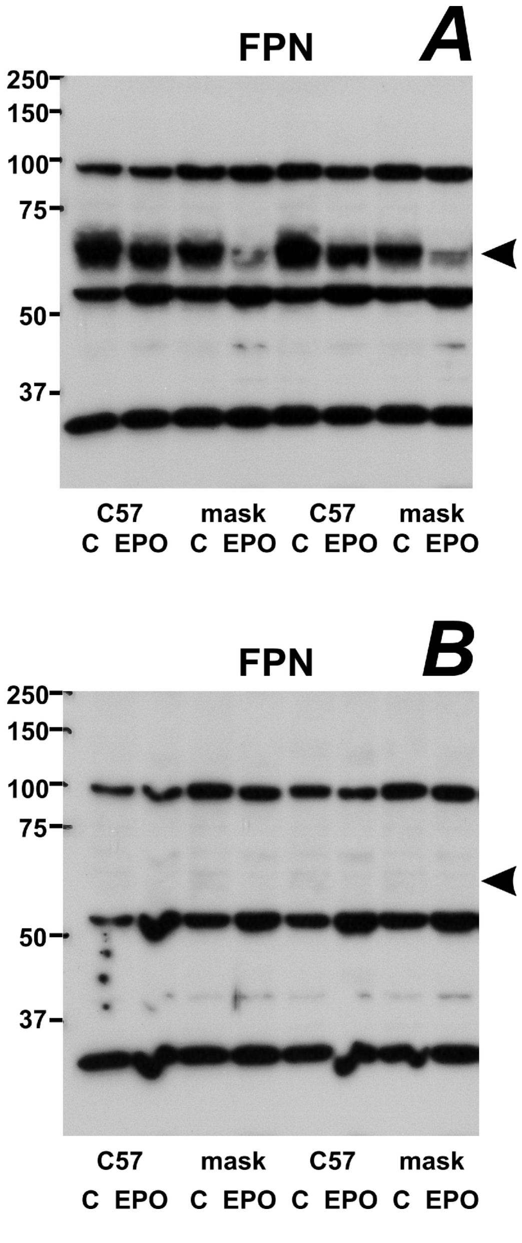

A: Detection of ferroportin (FPN) by Alpha Diagnostics International MTP1-A antibody in spleen homogenates heated for 5 min at 50°C prior to loading. Arrowhead denotes the FPN protein bands.

B: Attempt to detect FPN in similar samples heated for 10 min at 90 °C. No FPN bands are seen at the expected position (arrowhead) after heating the samples at higher temperature.
