# Supplementary material for: Effect of erythropoietin administration on proteins participating in iron homeostasis in Tmprss6-mutated mask mice
Source: PLoS One. 2017 Oct 26;12(10):e0186844. doi: 10.1371/journal.pone.0186844 (PMC5658091; doi:10.1371/journal.pone.0186844)
Supplement: S8 Fig — (PDF) [file pone.0186844.s012.pdf]

S8 Fig. Immunoblotting of FPN in spleen homogenates from C57BL/6 and *mask* mice

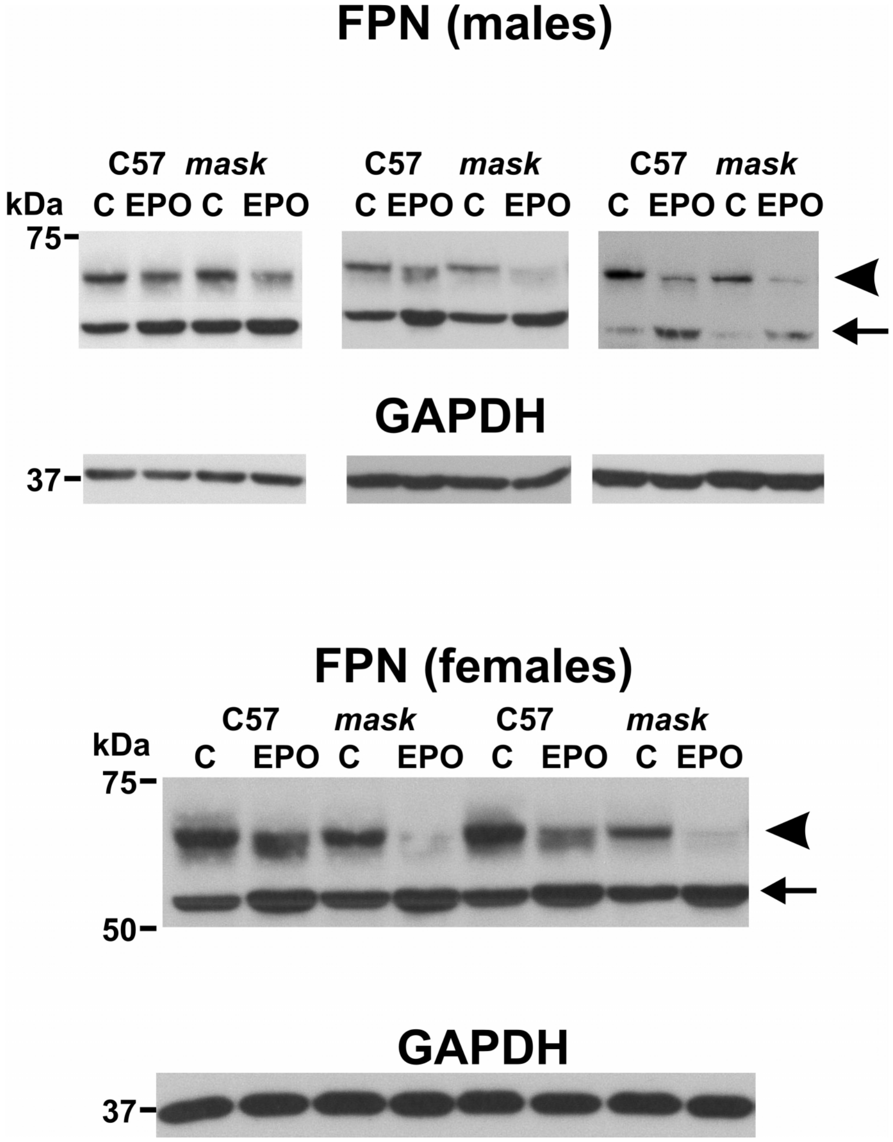

Ferroportin (FPN) protein content was determined by in spleen homogenates from PBS-treated (C) and EPO-treated (50 IU/mouse daily for four days) C57BL/6 (C57) and *mask* mice. Arrowhead denotes the ferroportin protein band used for densitometry quantification; arrow indicates a non-specific band at approximately 52 kDa. GAPDH is used as loading control. Signal intensities from these immunoblots, as well as the signal intensities from blots depicted in Fig. 5, are analyzed in S3 Table.
